# Experimental physiology Special Issue: ‘Mechanotransduction, muscle spindles and proprioception’

**DOI:** 10.1113/EP093067

**Published:** 2025-07-26

**Authors:** Stephan Kröger

**Affiliations:** ^1^ Department of Physiological Genomics, Biomedical Center Ludwig‐Maximilians‐Universität Planegg‐Martinsried Germany

This Special Issue of *Experimental Physiology* contains a collection of 11 exquisite articles most of which are based on oral presentations presented at the second meeting on ‘Mechanotransduction, Muscle Spindles and Proprioception’, which took place in the halls of the Siemens Foundation in Munich in July 2024. The participants included more than 30 speakers from Taiwan, Canada, Australia, the United States and Israel as well as from all over Europe and the UK (Figure 1). Many of the speakers had already presented in the first, & meeting on this topic in Munich in 2022 (Kröger, 2024). In this Editorial I would like to first briefly introduce the topic of the meeting before putting the individual articles into perspective.

The activity of almost every muscle is accompanied by sensory feedback informing the brain about the speed, direction and force of the movement. The interpretation of this information by the central nervous system (CNS) generates our sense of proprioception, which is essential for the execution of every voluntary movement, for perceiving the position of our body and limbs in relationship to one another and to our surroundings, and for posture and dexterity (Proske & Gandevia, 2012). However, proprioception has many more functions, including, for example, the alignment of the spine (Blecher et al., 2017) and healing of fractured bones (Blecher et al., 2017). The responsiveness of our proprioceptive information is also subject to cognitive and emotional factors (Ackerley et al., 2017). Moreover, proprioceptive information endows us with a sense of self‐awareness – an important aspect particularly for amputees. Correspondingly, dexterity and the rejection rate are significantly improved in amputees if the prosthesis is equipped with devices that provide sensory feedback equivalent to proprioceptive information (Raspopovic et al., 2021).

Although proprioception is an accomplishment of an integrative system which processes information from a combination of peripheral sensory inputs including muscle length and tension, joint angle, and skin stretch (Macefield & Knellwolf, 2018), by far the most influential components are muscle spindles (Matthews, 2015). Embedded in almost every skeletal muscle, these primary proprioceptive sensory organs constantly relay information about muscle tone and length to the thalamus and then via the dorsal (posterior) column–medial lemniscal system to the CNS (Kröger & Watkins, 2021; Marasco & de Nooij, 2023; Proske & Gandevia, 2012). The processing and integration of this information in the CNS allows the precise determination of the spatial position and motion of the body and limbs in space, a process crucial for motor control, voluntary movement, posture and a stable gait. Moreover, the human body can initiate corrective postural adjustments through appropriate locomotor commands (Ernst & Banks, 2002). However, the mechanism that transforms the proprioceptive sensory feedback information into a dynamic body percept is still only poorly understood.

Despite their enormous importance for movement control, the sense of proprioception and the function of muscle, spindles are heavily under‐represented – if not ignored – in many modern neuroscience or sensory physiology textbooks. For example, there is only about a single page devoted to proprioception and muscle spindles in the textbook by (Wolfe et al., 2012) and only two pages in the textbook by (Yantis, 2014). This special issue of *Experimental Physiology* entitled ‘Mechanotransduction, Muscle Spindles and Proprioception’ with its collection of eloquently written articles was solicited in part with the intention to ignite future research and to bring the topic to the attention of scientists, particularly to those in an early phase of their career.

The meeting started with the ‘*Experimental Physiology* Distinguished Lecture’ by Manuel Hulliger, who gave a historical and personal account of Peter B.C. Matthews's (1928–2020) contributions to the muscle spindle and proprioception field, summarizing many years of research on the structure and function of the mammalian muscle spindle, its intrafusal muscle fibres and their innervation by static and dynamic motor neurons (Matthews, 2015). Peter Matthews is clearly one of the giants on whose shoulders modern muscle spindle and proprioception researchers are standing. Moreover, during his days, muscle spindles were at the forefront of neuroscience. Many fundamental principles of neuroscience were discovered using this sense organ (Kröger, 2024).

Computational models help to understand how feedback from multiple proprioceptive sensory organs signal muscle state variables in order to control movement. In this Special Issue, Stephens and colleagues, using novel computational approaches, demonstrate how combinations of group Ia and II muscle spindle afferent feedback can allow for tuned responses to force and the rate of force (or length and velocity) and how combinations of muscle spindle and Golgi tendon organ feedback can parse external and self‐generated force (Stephens et al., 2025). These models suggest that muscle spindle feedback may be used to monitor and control muscle forces in addition to length and velocity, and – when combined with tendon organ feedback – can distinguish self‐generated from externally imposed forces. Since these models incorporate feedback from different sensory afferent types, they analyse muscle propriosensors as an integrated population of stimuli rather than independently.

Next to the eye and the inner ear, muscle spindles are one of the most complex peripheral sensory organs. It is therefore interesting to investigate the evolutionary origin of these structures. In a review published in this Special Issue, Banks and Proske have worked their way through the available literature to search for the presence and structure of muscle spindles in many different species (Banks & Proske, 2025). Using a comparative morphological approach, they propose that the need for spindles evolved as a result of the transition from an aquatic to a terrestrial habitat. With the presence of a capsule, one or more intrafusal fibres, and sensory and motor innervation being the defining characteristics of muscle spindles, they also suggest that during evolution, muscle spindles evolved at least twice. They appeared first in early amniotes when they became fully terrestrial and then again separately, independently and likely much later in anurans when they began to inhabit a terrestrial environment Banks & Proske, 2025). This would suggest the possibility that muscle spindles are an example of convergent evolution in two disparate species. It will be interesting to test this hypothesis by a similar analysis of the evolution of other mechanosensitive organs like Merkel cells or with the evolution of key molecules required for mechanotransduction, like the PIEZO channels.

How can we quantify proprioception non‐invasively and with high precision in humans? The review by Uwe Proske in this Special Issue (Proske, 2025) summarizes current concepts to measure position sense in humans, focusing particularly on three commonly used methods (two‐arm matching, one‐arm pointing and one‐arm repositioning; Roach et al., 2023). Under experimental conditions, all methods are performed by blindfolded subjects and can be easily quantified. It is unknown if the sense of movement and/or the sense of position are assayed to the same extent by the three methods. This appears important since the sense of movement is distinct from the sense of position (McCloskey, 1973). The sense of movement is believed to be generated by the primary endings of spindles, while both primary and secondary endings contribute to position sense (Banks et al., 2021). The review also addresses the question of whether these three methods are based on information provided specifically by muscle spindles. Using the presence of thixotropic errors occurring only in the position signal in response to conditioning voluntary contractions of muscles, evidence is provided for spindles contributing to position sense with all three methods – however to a different extent. Thus, in a clinical setting, each of the three methods has its own advantages and disadvantages. Clearly, analysing position sense is more complex than previously anticipated. Moreover, the different dependences of the three methods on muscle spindle activity might be taken as argument for the existence of more than one position sense.

**FIGURE 1:**
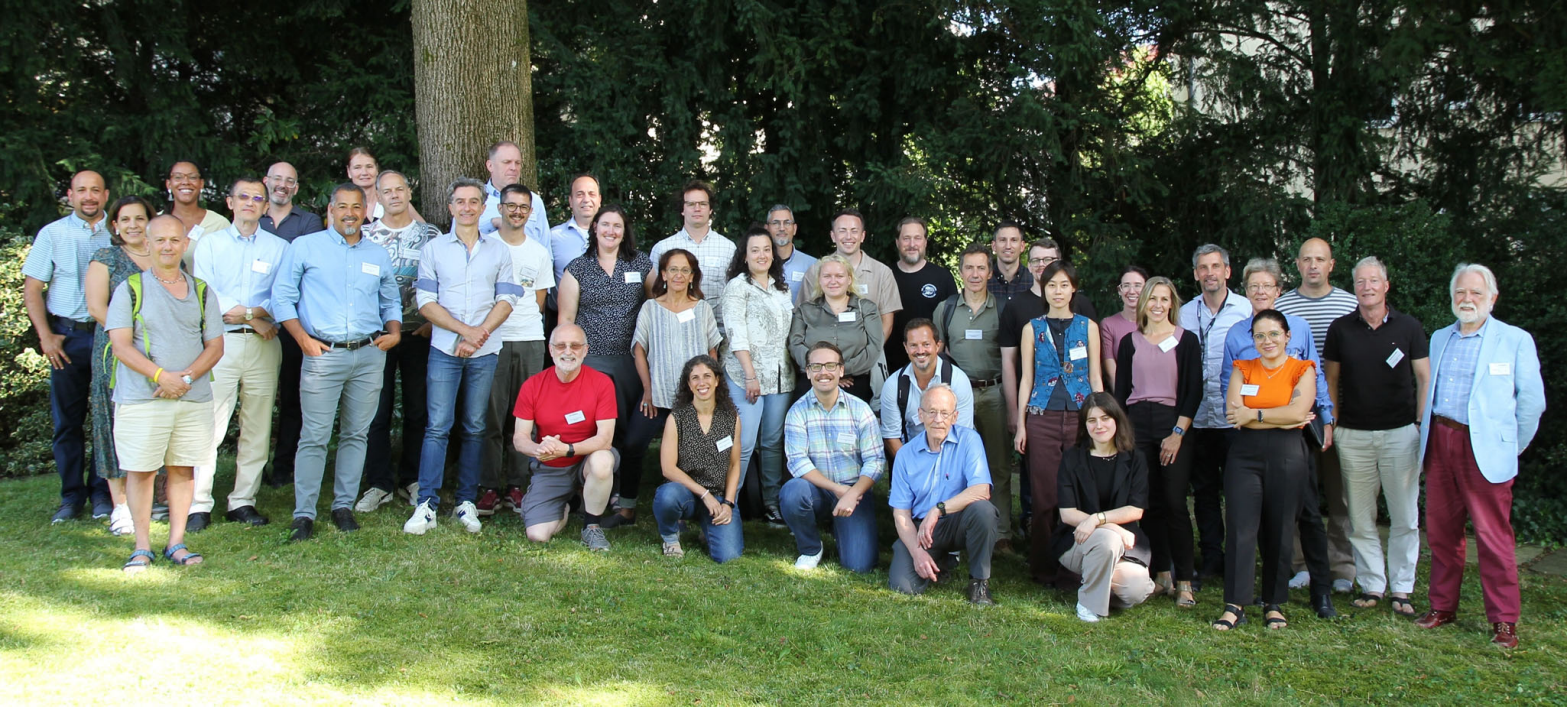
Group picture of the participants of the 2nd meeting on ‘Mechanotransduction, muscle spindles and proprioception’ held in Munich July 2024.

Proprioceptive judgements can be divided into two broad categories: low‐level and high‐level. Low‐level judgements of limb position require a person to detect, discriminate or match the position of a body part, whereas high‐level judgements require a person to report the position of an unseen body part relative to the external world. In a publication in this Special Issue, Gandevia and colleagues investigated if muscle thixotropy – the influence of recent contraction or stretch on the passive properties of a muscle – influences both the accuracy of high‐level judgements of limb position and the degree to which these judgements drift over time (Gandevia et al., 2025). Participants made visual judgements about the perceived position of their hidden index finger after their elbow muscles had been conditioned with a flexion or extension contraction, or after a series of large passive elbow movements. They report that there was little to no effect of either contraction type on drift in perceived index finger position, suggesting that muscle thixotropy has only a minimal effect on high‐level proprioceptive judgements. This also suggests that muscle spindle signals do not dominate the central, cross‐modal transformations of sensory information that are required for high‐level proprioceptive judgements.

Estimates have suggested that when healthy adults stand on a firm surface, 70% of the sensory contribution to postural stability is from proprioception, 20% from vestibular feedback, and only 10% from vision (Peterka, 2002), demonstrating proprioception as the dominant sensory resource for achieving postural stability. Accordingly, an impaired function of proprioception results in a decline of balance control, leading to an increased risk of falls. Falls have become the leading cause of accidental death among older individuals. About one‐third of the population aged 65 years and over will fall in a year, rising to more than half of those aged 80 years and older; a quarter of those falling will suffer a life‐changing injury (Centers for Disease Control and Prevention, 2023; https://www.cdc.gov/falls/data‐research/; Lamb et al., 2024). An estimated 684,000 fatal falls occur globally each year, making it the second leading cause of unintentional‐injury death, after road traffic injuries. In addition, approximately 37.3 million falls occur each year that are severe enough to require medical attention (https://www.who.int/news‐room/fact‐sheets/detail/falls). In Europe, the health and social care costs of falls are approximately €25 billion each year and will rise as populations age (Joint Declaration issued by the European Stakeholders Alliance for Active Ageing through Falls Prevention (2015); https://go.nature.com/3S3iKQo), demonstrating the severe socio‐economic burden of falls to the health system (Muir et al., 2010). The maintenance of a good balance in standing via the proprioceptive system is therefore an important aspect of health, in particular for elderly persons – and information on the status of the proprioceptive system is of paramount importance to prevent falls.

In the current Special Issue, the paper by Xie and colleagues carefully investigated the contribution of proprioception to balance control in ageing persons. By blindfolding and applying mastoid vibrations, the ageing‐related sensory deteriorations particularly of the proprioceptive system could be analysed (Xie et al., 2025). They show that mastoid vibration was able to simulate a vestibular‐disrupted environment, increasing the magnitude and irregularity of centre of gravity displacement. When standing with mastoid vibration applied, older adults demonstrated poorer balance control than young adults. They attribute a high risk of imbalance to ageing‐related proprioceptive and vestibular deteriorations even in healthy older adults (Xie et al., 2025).

Proprioception starts with mechanotransduction, that is, the transformation of a mechanical stimulus into a change of the receptor potential in the terminals of proprioceptive sensory neurons within the muscle spindles. There is no doubt that the key mechanically gated ion channel in humans and rodents is the PIEZO2 channel (Chesler et al., 2016; Woo et al., 2015), but other ion channels might modulate the receptor potential initially generated by PIEZO2. The receptor potential is subsequently transformed into a series of action potentials with the stimulus intensity being directly proportional (within the linear range) to the frequency of the action potentials. This so‐called ‘rate coding principle of stimulus intensity’ applies to sensory information processing in the entire nervous system. This discovery was awarded with the Nobel Prize for Physiology or Medicine to E.D. Adrian and C.S. Sherrington in 1932.

The presence of extracellular calcium is crucial for the normal function of every cellular component of muscle tissue, including extra‐ and intrafusal muscle fibres and their neuronal innervation. In the muscle spindle sensory terminal, extracellular calcium has been shown to be essential for secretion and uptake of glutamate‐containing synaptic‐like vesicles (Bewick et al., 2005). Moreover, while the generation of the receptor potential exclusively relies on sodium, the presence of a residual stretch‐activated calcium current has been reported in the absence of extracellular sodium (Hunt et al., 1978). Interestingly, removal of calcium from the extracellular medium abolishes stretch‐evoked action potentials (Bewick et al., 2005; Kruse & Poppele, 1991) suggesting an important influence of calcium on action potential generation or propagation. However, the calcium channel(s) involved, and their mechanism(s) of action are unknown. In a study published in this Special Issue, the lab of Guy Bewick aimed at identifying the calcium channels involved in muscle spindle mechanotransduction and action potential propagation (Simon et al., 2025). They used specific antagonistic and agonistic calcium channel toxins in adult rat lumbrical muscle to investigate their effect on stretch‐evoked muscle spindle afferent discharge. They also used live spindle sensory terminal labelling with the dye FM1‐43 to monitor synapse‐like vesicle recycling. They report that inhibitors of voltage‐gated L‐type channel blockers inhibited FM1‐43 release, while TRPV4 (transient receptor potential, vanilloid, type 4) channel blockers entirely inhibited FM1‐43 uptake. Moreover, multiple potassium channels gated by voltage‐activated (L‐ and P/Q type) calcium channels regulate action potential firing rates of afferent proprioceptive sensory neurons (Simon et al., 2025). In a ‘Viewpoint’ in this Special Issue, Vaughan Macefield provides more information regarding the importance of these findings (Macefield, 2025).

Nerve regeneration is associated with plasticity of sensory neurons such that even muscle afferents directed to the skin form mechanosensitive receptive fields appropriate for the new target. When proprioceptive or touch‐sensitive afferent neurons are severed, they regain mechanosensitivity within hours after axotomy (Koschorke et al., 1994), a finding indicating that the molecules required for mechanosensitivity are already present in regenerating sensory axons. One molecular component of mechanosensitivity is the integral membrane protein stomatin like protein‐3 (STOML3). This protein is an essential component of the mechanotransduction complex in many mechanoreceptors (Wetzel et al., 2007, 2017). It significantly increases the sensitivity of the mechanosensitive PIEZO2 channels (Chakrabarti et al., 2024; Poole et al., 2014), essential for many mechanoreceptors and proprioceptors including human (Chesler et al., 2016) and murine (Woo et al., 2015) muscle spindles. Indeed, in either *stoml3* or *Piezo2* mutant mice, around 40% of cutaneous myelinated sensory afferents completely lack mechanosensitivity (Ranade et al., 2014; Wetzel et al., 2007, 2017). However, unlike PIEZO2‐deficient humans and mice, *stoml3*‐deficient mice do not have proprioceptive deficits (Ranade et al., 2014; Wetzel et al., 2007). In a publication in this Special Issue, the lab of Gerry Lewin asked if STOML3 is required for functional and anatomical plasticity following peripheral nerve regeneration (Haseleu et al., 2025). They used a cross‐anastomosis model in mice in which the medial gastrocnemius nerve (a pure muscle nerve) was redirected to innervate hairy skin previously occupied by the sural nerve. Recording from muscle afferents innervating the skin, they observed that in mice lacking STOML3, muscle afferents largely failed to form functional mechanosensitive receptive fields, despite making anatomically and somatotopically appropriate endings in the skin. Interestingly, in the spinal cord, the terminals of muscle afferents now innervating the skin in *stoml3* mutant mice terminated in a somatotopically organized fashion in dorsal horn laminae. Thus, muscle‐derived afferents confronted with a new target in the skin can exhibit substantial structural plasticity. The substantial loss of stimulus‐evoked activity in most of the redirected muscle afferents in the skin of *stoml3* mutant mice did not prevent these afferents from displaying similar structural plasticity to controls (Haseleu et al., 2025). This identifies STOML3 as the first molecule, required for functional plasticity following peripheral nerve injury in vivo. The molecular mechanism of STOML3 remains to be determined, in particular if it acts via its effect on mechanotransduction or via a different pathway.

A method to directly assay human muscle spindle afferent responses to stretch is microneurography (Vallbo, 2018). It involves inserting a fine, sterile tungsten microelectrode into a peripheral nerve (e.g. peroneal, or radial nerve) to measure the activity of afferent or efferent nerve fibres in real time. Using this method, the lab of Vaughan Macefield characterized for the first time the firing properties of muscle spindle endings in the intrinsic muscles of the foot and of cutaneous mechanoreceptors in the sole during unsupported standing (Knellwolf et al., 2025). The responsiveness of muscle spindles in the short muscles of the foot to stretch and related joint movements was similar to that of spindles located in the intrinsic muscles of the hand. Most spindle afferents (∼70%) were silent when the foot was unloaded but fired tonically during standing, with their discharge rate encoding changes in the centre of pressure. Most cutaneous afferents responded only during contact and incidental adjustments in posture. They conclude that spindle endings in the muscles of the foot, in addition to tactile afferents from the sole, provide proprioceptive information during standing. Both systems contribute to the maintenance of upright posture.

Like any other sense, proprioception is also subject to illusions. In a study published in this Special Issue of *Experimental Physiology*, the Mathis lab modelled classic proprioceptive illusions in which tendon vibrations lead to biases in estimating body position using deep‐learning models of the ascending proprioceptive pathway (Perez Rotondo et al., 2025). Task‐driven models that have been trained to infer the state of the body from distributed sensory muscle spindle inputs (primary and secondary afferents) but not trained with illusion experiments and simulated muscle–tendon vibrations were used. Interestingly, these task‐driven models were susceptible to proprioceptive illusions, with the magnitude of the illusion depending on the vibration frequency, demonstrating that primary proprioceptive afferents alone are sufficient to account for these classic illusions.

Gamma motor neurons form neuromuscular junctions in the polar regions of the intrafusal fibres and control the sensitivity of the muscle spindles to stretch by regulating the length of the equatorial muscle spindle sensory region (Banks, 1994). γ‐Motoneurons differ from α‐motoneurons (innervating extrafusal fibres) in their electrical, cellular and molecular properties as well as in their development (Blum et al., 2021; Kanning et al., 2010; Khan et al., 2022; Liau et al., 2023; Manuel & Zytnicki, 2011). Moreover, γ‐motoneurons are spared in at least two neuromuscular diseases, amyotrophic lateral sclerosis and spinal muscle atrophy, and spared γ‐motoneuron activity might contribute to disease progression (Lalancette‐Hebert et al., 2016; Powis & Gillingwater, 2016). In a publication in this Special Issue, Wilkinson and colleagues compared mice which express channelrhodopsin‐2 in both types of motoneurons (cholineacetyltransferase‐positive neurons) to mice expressing channelrhodopsin‐2 selectively in γ‐motoneurons (Npas1‐positive neurons; Karekal et al., 2025). Use of an *ex vivo* muscle–nerve preparation (Wilkinson et al., 2012) allowed them to place a light guide directly on the nerve while simultaneously recording single unit muscle spindle afferent firing with an extracellular electrode. This provides a functional readout of both α‐ and γ‐motoneuron activity, with a twitch contraction indicating α‐motoneuron stimulation and increased firing rates of muscle spindle afferents indicating γ‐motoneuron activity. This optogenetic stimulation protocol has the potential to become an exciting tool to selectively manipulate γ‐motoneuron activity and to investigate γ‐motoneuron function during voluntary movement during normal behaviour and disease (Karekal et al., 2025).

In summary, the collection of articles in this Special Issue of *Experimental Physiology* provides a valuable resource for researchers and clinicians interested in proprioception and the structure, function and pathology of muscle spindles. By integrating original research with in‐depth reviews, this issue certainly enhances our understanding of the role of proprioception in motor control. My hope is that these publications will also inspire further investigations by fostering collaboration between researchers, clinicians and educators. This should bring muscle spindles and proprioception to the attention of those writing textbooks so that younger scientists can be introduced to and get excited about this area of research.

It only remains to thank all those involved in helping to initiate and organize the meeting. Most importantly, I would like to thank Nellie Kwabla and Jürgen Schultheiss without whose hard work, dedication and co‐ordinating activities the conference would not have been successful. The conference took place in the beautiful settings of the Carl Friedrich von Siemens Foundation (represented by Mr De Gasperi) who not only provided us with the technical equipment but also with exquisite food. The garden was a perfect site for many discussions during the breaks and many collaborations were established during the discussions in this exquisite location. I would also like to thankfully acknowledge the financial support from the German Research Foundation (DFG; KR1039/22‐1) and from our industrial sponsor, Nanion Technology. Finally, I would like to thank Bob Banks and Guy Bewick together with the team from *Experimental Physiology*, especially Joshua Hersant, for their unstinting support and patience during the compilation of the papers in this Special Edition. The journal *Experimental Physiology* also provided generous financial support for an ‘*Experimental Physiology* Distinguished Speaker Award’, an ‘*Experimental Physiology* Young Scientist Award’ and an ‘*Experimental Physiology* Travel Award’, which were gratefully received by the respective recipients.

## AUTHOR CONTRIBUTIONS

Sole author.

## CONFLICT OF INTEREST

No competing interests declared.

## FUNDING INFORMATION

The author received funding for the meeting from the Deutsche Forschungsgemeinschaft (DFG) with the Grant Reference Number KR1039/22‐1.
